# Migraine and restless legs syndrome are associated in adults under age fifty but not in adults over fifty: a population-based study

**DOI:** 10.1186/s10194-015-0554-0

**Published:** 2015-08-13

**Authors:** Soo-Jin Cho, Yun Kyung Chung, Jae-Moon Kim, Min Kyung Chu

**Affiliations:** Department of Neurology, Dongtan Sacred Heart Hospital, Hallym University College of Medicine, Hwaseong, Korea; Department of Occupational and Environmental Medicine, Sacred Heart Hospital, Hallym University College of Medicine, Anyang, Korea; Department of Neurology, Chungnam National University, College of Medicine, Daejeon, Korea; Department of Neurology, Sacred Heart Hospital, Hallym University College of Medicine, 896 Pyungchon-dong, Dongana-gu, Anyang, Gyeonggi-do 431-070 Korea

**Keywords:** Comorbidity, Cross-sectional study, Epidemiology, Headache, Migraine, Restless legs syndrome

## Abstract

**Background:**

Recent studies have shown an association between migraine and restless legs syndrome (RLS). However, migraine prevalence peaks from the 20s to 40s whereas RLS prevalence peaks after the 50s. Despite this, reports on how migraine and RLS may be associated by age is limited. Therefore, the purpose of this study is to investigate the comorbidity between migraine and RLS according to age.

**Methods:**

We selected a stratified random population sample of Koreans aged 19 to 69 years and evaluated them with a 60-item semi-structured interview designed to identify RLS, headache type, and clinical characteristics of migraine. To assess the association between migraine and RLS according to age, we divided participants into 5 age groups (19–29, 30–39, 40–49, 50–59, and 60–69 years) and analysed each group.

**Results:**

Subjects with migraine showed an increased RLS prevalence in the 19–29 (Odds ratio [OR] = 6.6, 95 % confidence interval [CI] = 1.2–36.8) and 40–49 (OR = 6.7, 95 % CI = 1.5–33.5) age groups compared to non-headache controls but failed to showed a significant association in the 50–59 (OR = 1.1, 95 % CI = 0.2–5.6) and 60–69 (OR = 0.4, 95 % CI = 0.1–4.0) age groups. Migraineurs with 1–10 (12.5 %, OR = 2.0, 95 % CI = 1.3–3.2, p = 0.003) and >10 (12.5 %, OR = 2.5, 95 % CI = 1.0–5.6, p = 0.038) attacks per month showed an increased RLS prevalence compared to migraineurs with <1 attack per month (2.1 %). Subjects with non-migraine headaches showed an increased odds for RLS (OR = 1.8, 95 % CI = 1.3–2.7) compared to non-headache controls. There was no significant difference (9.1 % vs. 6.9 %, p = 0.339) in the RLS prevalence between migraineurs and non-migraine headache subjects.

**Conclusions:**

Our results suggest that migraine and RLS are differently associated according to age.

**Electronic supplementary material:**

The online version of this article (doi:10.1186/s10194-015-0554-0) contains supplementary material, which is available to authorized users.

## Background

Migraine is a common chronic neurological disorder affecting 6–20 % of the general population, with a female-to-male ratio of 2–3:1 [1–7]. Migraine prevalence peaks from the 20s to 40s which is the most productive age in sufferers’ lives [1, 2, 4, 8]. It typically presents as recurrent headache attacks, with associated symptoms including hypersensitivity. It also causes substantial levels of disability in daily life by chronically disrupting work, personal, and family activities. The World Health Organization lists migraine as the seventh leading cause of disability [8].

Epidemiological and clinical studies have shown that migraine is comorbid with a number of diseases including cardiovascular disorders, depression, anxiety, and other pain disorders [1, 9, 10]; and this comorbidity may increase the affected sufferer’s level of disability [11, 12]. Migraine may also share pathophysiological mechanisms with comorbid conditions [12]. However, migraine and its comorbid conditions are usually underdiagnosed and undertreated [13].

Restless legs syndrome (RLS) is a common sensory-motor disorder with a similar female predominance. The 1-year prevalence is estimated at 2–15 %, and the approximate female-to-male ratio is 2:1 [14, 15]. The International Restless Legs Study Group (IRLSSG) has defined diagnostic criteria for RLS according to its typical clinical presentation [14]. Unlike migraine, RLS prevalence increases with age and peaks after the 50s [15–22].

Epidemiological and clinical studies have shown an association between migraine and RLS [23–28]. However, the difference in the age-dependent prevalence of the 2 disorders suggests that the association may differ by age. However, the association of migraine and RLS in different age groups has yet not been determined. We therefore sought to investigate the association between migraine and RLS according to age groups using Korean Headache-Sleep Study data, a nationwide population-based study regarding headache and sleep disorders. A secondary outcome was to evaluate RLS prevalence according to the frequency of migraine headaches in order to corroborate the association of 2 disorders.

## Methods

### Study population

The Korean Headache-Sleep Study was a nationwide, cross-sectional survey of headache and sleep in the Korean population in adults aged 19–69 years. The study design and methods were previously described in detail [29, 30]. Briefly, we adopted a 2-stage systematic random sampling method in all Korean territories except Jeju-do proportional to population distribution and sampled 2,695 individuals. Subjects were stratified according to age, gender, and occupation. To minimized interest bias, we informed candidates that the survey topic was “social health issue” rather than headache. Trained interviewers conducted structured interviews, using a questionnaire to diagnose headache and sleep disorders including RLS by door-to-door visit and face-to-face interview. The interview included questions on the symptoms of headache and RLS. All interviewers were employed by Gallup Korea and had previous social survey interviewing experience. The interviewers were not medical personnel. The study was undertaken from November 2011 to January 2012, and was approved by the institutional review board/ethics committee of Hallym University Sacred Heart Hospital and was performed in accordance with the ethical standards laid down in the 1964 Declaration of Helsinki and its later amendments. Written informed consent was obtained from all participants.

### Migraine assessment

We diagnosed migraine using a questionnaire (Additional file 1). The questionnaire established a headache profile, which was designed to comply with the second edition of the International Classification of Headache Disorders (ICHD-2). We investigated severity of headache based on effect of daily activity (mild, moderate and severe) and using visual analogue scale. Migraine was diagnosed based on the ICHD-2 criteria for migraine without aura (code 1.1) [31]. We did not attempt to separately diagnose migraine with aura and migraine without aura. As such, both were included in the present study. The questions used to diagnose migraine were previously found to have 75.0 % sensitivity and 88.2 % specificity, by comparing the diagnoses from the survey with doctors’ diagnoses obtained from an additional telephone interview. The validation process was described in one of author’s (MKC) previous article in detail [3].

### Non-migraine headache assessment

If a participant responded positively to the question, ‘In the past year, have you had at least 1 headache lasting more than 1 min?’ and was not diagnosed as having migraine, she or he was diagnosed as having non-migraine headaches.

### RLS assessment

A diagnosis of RLS was assigned if a participant responded positively to all 4 questions based on the IRLSSG criteria published in 2003: (1) ‘Do you have unpleasant sensations such as crawling or pain in your legs combined with an urge or need to move your legs?’ (2) ‘Do these feelings/symptoms occur mainly or only at rest?’ (3) ‘Does movement improve these unpleasant sensations?’ (4) ‘Are these symptoms worse in the evening or at night than in the morning?’ [14].

### Covariate information assessment

We assessed the socioeconomic and demographic characteristics of participants by using a questionnaire. Sleep quality was assessed by the Pittsburgh Sleep Quality Index (PSQI) questionnaire considering total scores of >5 as ‘poor sleep quality’. The Korean translation of PSQI has previously been validated [32].

### Statistical analysis

We compared the clinical characteristics of migraine participants with RLS and migraine participants without RLS using Student’s *t*-test for continuous variables and the chi-square test for categorical variables. For comparing RLS prevalence among non-headache control, non-migraine headache, and migraine participants, we used the chi-square test.

We used logistic regression analysis models to evaluate the association between migraine and RLS and calculated odds ratios (ORs) and their 95 % confidence intervals (CIs). We conducted multivariable logistic regression analysis adjusting for demographic variables and sleep quality. Specifically, the multivariable model controlled for gender, size of residential area, educational level, and sleep quality (PSQI score >5).

For assessment of the association between migraine and RLS according to age, we divided our participants into 5 age groups (19–29 years, 30–39 years, 40–49 years, 50–59 years and 60–69 years) and conducted logistic regression analysis for each age group.

As with most survey sampling designs, non-response resulted in data missing from several variables. The data reported are based on the available data. Sample sizes of some variables diverge from the total sample size of 2,695 because of non-responses for that particular variable. Imputation techniques were not employed to minimise non-response effects [33].

In all statistical analyses, the significance level was 0.05, unless otherwise specified. The results were analysed using the Statistical Package for the Social Sciences 22.0 (SPSS 22.0; IBM, Armonk, NY, USA).

## Results

**Fig. 1:**
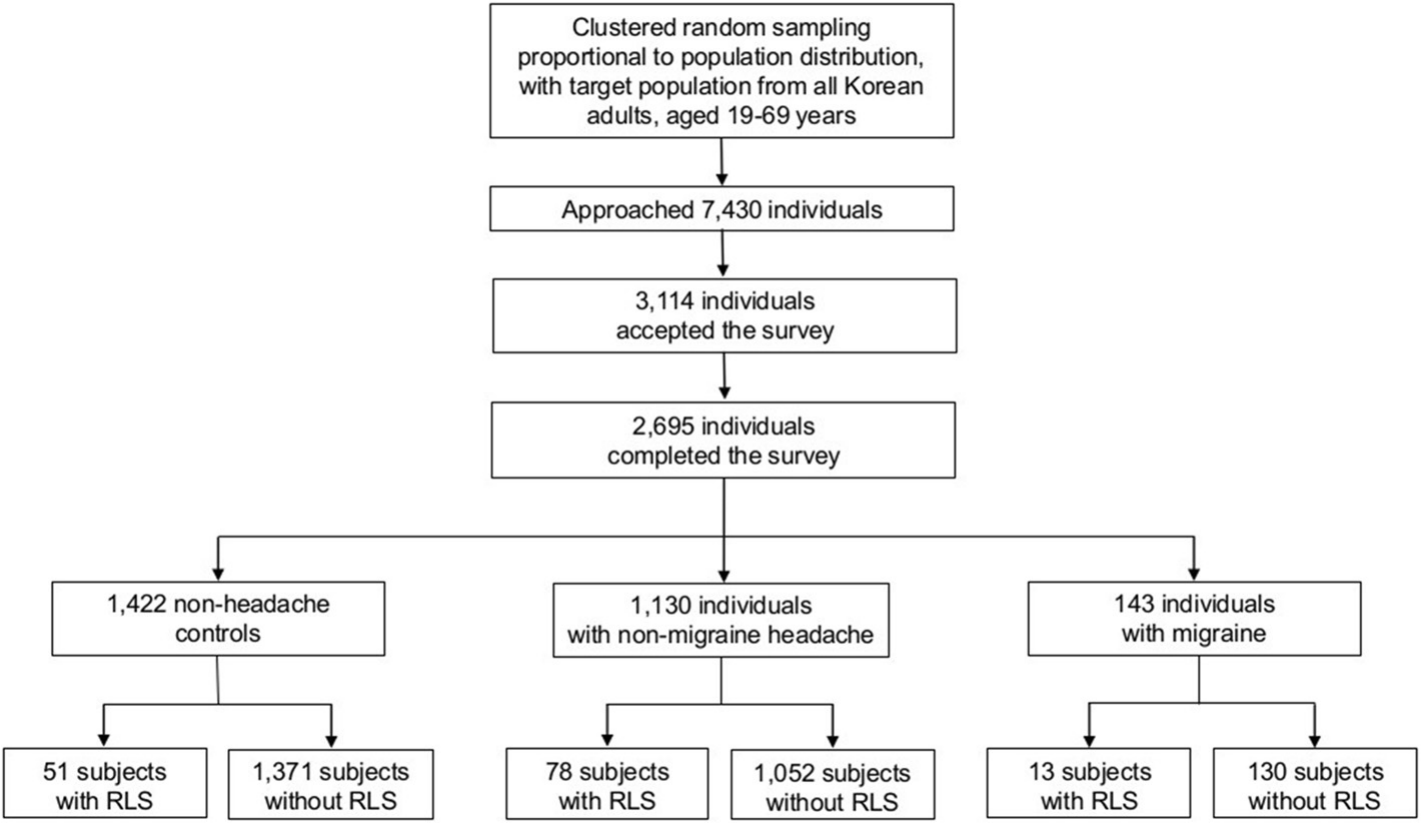
Flow chart depicting the participation of subjects in the Korean Headache-Sleep Study. RLS: restless legs syndrome

Our interviewers approached 7,430 individuals and 3,114 of them accepted the survey (rejection rate of 58.1 %). After 352 individuals suspended the interview, 2,695 subjects completed the survey (cooperation rate, 36.2 %; Fig. 1). The distributions of age, gender, size of residential area, and educational level were not significantly different from those of the general population of Korea (Fig. 1 and Table 1).

**Table 1:** Sociodemographic distribution of all survey participants, the total Korean population, and of cases identified as migraine and restless legs syndrome

		Sample number	Total population	p-value	Migraine		Restless legs syndrome	
		N (%)	N (%)		N	(% , 95 % CI)	N	(% , 95 % CI)
Gender								
	Men	1345 (49.3^a^)	16,357,919 (50.6)	0.42^b^	36	2.7, 1.8–3.5	56	4.1, 3.0–5.1
	Women	1350 (50.7^a^)	15,998,828 (49.4)		107	7.9, 6.5–9.4	86	6.4, 5.1–7.7
Age								
	19–29	542 (20.5^a^)	7,179,648 (22.2)	0.76^b^	25	4.5, 2.8–6.2	12	2.2, 1.0–3.4
	30–39	604 (21.9^a^)	7,767,141 (24.0)		42	7.0, 5.0–9.1	34	5.6, 3.7–7.4
	40–49	611 (23.1^a^)	8,012,377 (24.8)		39	6.5, 4.5–8.4	20	3.3, 1.9–4.7
	50–59	529 (18.9^a^)	5,737,344 (17.7)		22	4.1, 2.4–5.9	35	6.5, 4.4–8.7
	60–69	409 (15.6^a^)	3,660,237 (11.3)		15	3.9, 2.0–5.7	41	10.2, 7.3–13.1
Size of residential area								
	Large city	1,248 (46.3^a^)	1,5606,652 (48.2)	0.92^b^	76	6.1, 4.8–7.5	66	5.3, 4.1–6.5
	Medium-to-small city	1,186 (44.0^a^)	1,4106,687 (43.6)		48	4.0, 2.9–5.1	58	4.9, 3.7–6.1
	Rural area	261 (9.7^a^)	264,307 (8.2)		19	7.4, 4.2–10.6	18	6.8, 3.7–9.8
Educational level								
	Middle school or less	393 (14.9^a^)	6,147,782 (19.0)	0.75^b^	22	5.5, 3.3–7.7	41	10.5, 7.5–13.5
	High school	1,208 (44.5^a^)	14,172,255 (43.8)		60	5.0, 3.8–6.3	62	5.1, 3.8–6.3
	College or more	1,068 (39.6^a^)	1,2036,710 (37.2)		60	5.6, 4.3–7.0	38	3.6, 2.5–4.7
Total		2,695 (100.0^a^)	32,356,747 (100.0)		143	5.3, 4.5–6.2	142	5.3, 4.4–6.1

*CI* confidence interval

^a^Adjusted after age, gender, size of residential area and educational level

^b^Compared gender, age group, size of residential area, and educational level distributions between the sample of the present study and total population of Korea

### Prevalence of migraine and RLS

Of the 2,695 participants, 1,273 (47.2 %) subjects had had at least 1 attack of headache, and 143 subjects (5.3 %) met the ICHD-2 criteria for migraine. So, 1,130 subjects were classified as having non-migraine headaches and 1,422 subjects were classified as non-headache controls. One hundred forty two (5.3 %) subjects positively responded at all 4 questions from the IRLSSG criteria and were classified as having RLS (Table 1).

### RLS in subjects with migraine

Of 143 migraine subjects, 13 (9.1 %) were classified as having RLS. Of 1,130 non-migraine headache subjects and 1,422 non-headache control subjects, 78 (6.9 %) and 51 (3.6 %) subjects were classified as having RLS, respectively. A significantly greater prevalence of RLS was observed in both subjects with migraine (OR = 2.6, 95 % CI = 1.4–5.0, p = 0.003) and non-migraine headaches (OR = 2.1, 95 % CI = 1.4–2.9, p < 0.001) relative to non-headache control subjects. This pattern was consistent even after adjusting for sociodemographic variables (age, gender, educational level, and size of residential area) and sleep quality (PSQI score >5) (Table 2). There was no significant difference (9.1 % vs. 6.9 %, p = 0.339) in the RLS prevalence between migraineurs and non-migraine headache subjects.

**Table 2:** Prevalence and odds ratios of restless legs syndrome according to headache diagnosis

Age group, No of participants/ No. of RLS	Non-headache control, N = 1,422		Non-migraine headache, N = 1,130			Migraine, N = 143		
	No. of RLS (%)	OR	No. of RLS (%)	OR (95 % CI)	P-value	No. of RLS (%)	OR (95 % CI)	P-value
Aged 19–29, 542/12	4 (1.4)	Reference	5 (2.2)	1.3 (0.4–5.0)	0.701	3 (12.0)	6.6 (1.2–36.8)	0.029
Aged 30–39, 604/34	6 (2.0)	Reference	25 (9.3)	4.6 (1.8–12.0)	0.002	3 (7.1)	2.9 (0.6–13.4)	0.176
Aged 40–49, 611/20	3 (1.0)	Reference	13 (4.7)	3.7 (1.0–13.5)	0.046	4 (10.3)	6.7 (1.5–33.5)	0.016
Aged 50–59, 529/35	17 (5.6)	Reference	16 (7.8)	1.3 (0.6–2.7)	0.553	2 (9.1)	1.1 (0.2–5.6)	0.902
Aged 60–69, 409/41	21 (8.6)	Reference	19 (12.8)	1.3 (0.6–2.5)	0.506	1 (6.7)	0.4 (0.1–4.0)	0.459
Total, 2695/142	51 (3.6)	Reference	78 (6.9)	1.8 (1.3–2.7)^a^	0.002^a^	13 (9.1)	2.2 (1.1–4.3)^a^	0.018^a^

*CI* confidence interval, *OR* odds ratio, *RLS* restless legs syndrome

^a^Adjusted after sociodemographic variables and sleep quality

### RLS prevalence according to age

We conducted logistic regression analysis for RLS prevalence according to headache diagnosis in 5 age groups. Subjects with migraine showed increased ORs for RLS in the 19–29 age group (OR = 6.6 95 % CI = 1.2–36.8, p = 0.029) and the 40–49 age group (OR = 6.7, 95 % CI = 1.5–33.5, p = 0.016) compared to non-headache controls. For subjects with non-migraine headaches, the 30–39 age group (OR = 4.6, 95 % CI = 1.8–12.0, p = 0.002) and the 40–49 age group (OR = 3.7, 95 % CI = 1.0–13.5, p = 0.046) showed increased ORs for RLS compared to non-headache controls. Migraineurs and non-migraine headache subjects failed to show increased ORs for RLS compared to non-headache controls in the 50–59 and 60–69 age groups (Table 2). We further divided our participants into 2 age groups (aged <50 years and aged ≥50 years) and compared the association between migraine and RLS. Migraineurs showed an increased OR for RLS compared to non-headache controls in aged <50 years (OR = 6.9, 95 % CI = 2.9–16.2, p < 0.001) but failed to show in aged ≥50 years (OR = 1.1, 95 % CI = 0.3–3.9, p = 0.883). For non-migraine headache subjects, an increased OR for RLS compared to non-headache controls was observed in aged <50 years (OR = 3.9, 95 % CI = 2.1–7.4, p < 0.001) but not in aged ≥50 years (OR = 1.5, 95 % CI = 0.9–2.4, p = 0.099).

### RLS prevalence among migraineurs according to headache frequency

A significant increase in the prevalence of RLS was observed in both migraineurs with 1–10 attacks per month (12.5 %, OR = 2.0, 95 % CI = 1.3–3.2, p = 0.003) and migraineurs with >10 attacks per month (12.5 %, OR = 2.5, 95 % CI = 1.0–5.6, p = 0.038) relative to migraineurs with <1 attack per month (2.1 %) (Table 3).

**Table 3:** Prevalence and odds ratios of restless legs syndrome among migraineurs according to headache frequency

		RLS N (%)	OR (95 % CI)	*P*-value
Migraine	<1 attack per month, N = 47	1 (2.1)	Reference	Reference
	1–10 attacks per month, N = 80	10 (12.5)	2.0 (1.3–3.2)	0.003
	>10 attacks per month, N = 16	2 (12.5)	2.5 (1.0–5.6)	0.038

*CI* confidence interval, *OR* odds ratio, *RLS* restless legs syndrome

### Clinical characteristics of subjects having migraine with and without RLS

We investigated demographics, headache characteristics, associated symptoms, headache frequency per month, the visual analogue scale (VAS) score for pain intensity, and the Headache Impact Test (HIT-6) score of migraineurs according to the presence of RLS (Table 4). The only variable that showed a significant association with RLS was the HIT-6 score. The HIT-6 scores of migraineurs with RLS were significantly higher relative to migraineurs without RLS (59.1 ± 9.4 vs. 53.7 ± 9.2, p = 0.047).

**Table 4:** Clinical characteristics of migraine according to the presence of restless legs syndrome

		Migraine with RLS, N =13	Migraine without RLS, N =130	P-value
Women, N (%)		10 (76.9)	97 (74.8)	0.867
Age, mean ± SD		39.8 ± 12.6	41.5 ± 12.5	0.640
Frequency per month, mean ± SD		5.8 ± 8.6	3.7 ± 6.0	0.251
Headache characteristics	Unilateral, N (%)	9 (69.2)	72 (55.0)	0.323
	Pulsating, N (%)	11 (84.6)	97 (74.8)	0.432
	Moderate-to-severe intensity, N (%)	12 (92.3)	103 (78.6)	0.241
	Aggravation by movement, N (%)	8 (61.5)	92 (70.2)	0.516
Associated symptoms	Nausea, N (%)	11 (84.6)	114 (87.8)	0.742
	Vomiting, N (%)	6 (46.2)	49 (38.2)	0.573
	Photophobia, N (%)	8 (61.5)	76 (58.0)	0.806
	Phonophobia, N (%)	10 (76.9)	91 (69.5)	0.575
	Osmophobia, N (%)	7 (53.8)	61 (46.6)	0.616
Headache Impact test-6, mean ± SD		59.1 ± 9.4	53.7 ± 9.2	0.047
Visual analogue scale for pain intensity, mean ± SD		6.8 ± 2.2	6.1 ± 1.9	0.212

*RLS* restless legs syndrome, *SD* standard deviation

## Discussion

The key findings of the present study are as follows: 1) the prevalence of migraine and RLS in the Korean population were both 5.3 %; 2) RLS was more prevalent in migraineurs and subjects with non-migraine headaches when compared to non-headache control subjects in the 19–49 age groups but not in the 50–69 age groups; 3) the RLS prevalence was higher among migraineurs with >1 headache attacks per month compared to migraineurs with ≤1 headache attacks per month.

The 1-year migraine prevalence rate (5.3 %) in the present study was somewhat lower than that previously observed in Western countries (9–25 %) [34]. However, the migraine prevalence rate in the present study was similar to those observed in previous studies in Korea and other Asian countries [3, 35]. The 1-year prevalence of migraine ranges from 4.7 % to 9.1 % in most studies of Asian countries, which is somewhat lower than that recorded in studies of European and North American countries [35]. As in most migraine epidemiological studies, migraine prevalence peaked in the 30–49 age groups and decreased in the 50–69 age groups in the present study [1, 3, 5–7]. These findings may be attributed to hormonal effects on migraine in women [36].

RLS prevalence (5.3 %) in the present study was similar to those in previous studies in Korea and other Asian countries, ranging from 1.8 % to 8.3 % [15–17, 19, 22]. Like migraine, RLS prevalence in Asian populations was reported to be somewhat lower than that observed in Western populations [15, 24]. The wide range of RLS prevalence values reported in previous studies may be explained by differences in ethnicity, cultural background, survey methods, and assessment tools. Similar to previous epidemiological studies for RLS, the prevalence increased with age in the present study. The similarities in the prevalence of migraine and RLS between previous studies and the present study suggest that our study properly reflects the actual prevalence of each in the Korean population.

Over the past years, a number of epidemiological and clinical studies have reported an association between migraine and RLS [23–25, 27, 28]. In the present study, a similar association between migraine and RLS was found in the form of a higher RLS prevalence among migraineurs compared to non-headache controls. We further investigated the association between migraine and RLS in the different age groups and found that migraine and RLS showed a significant association in adults aged 19–29 and aged 40–49, but not in those aged 50–69 (Table 2). Among aged 30–39, a trend towards an association was observed.

The different associations observed between migraine and RLS according to age could be explained by several factors. First, difference in pathophysiological mechanisms according to age in migraine and RLS may explain this lack of a significant association in the elderly population. Dopamine and iron dysregulation have been proposed to be mechanisms both for migraine and RLS [37, 38]. There were some evidences of different dopamine or iron dysregulation in migraine and RLS according to age. Iron deposition in deep brain nuclei was significant among migraineurs aged <50 years than age-matched controls, but not among migraineurs aged ≥50 years [39]. Striatal D2 dopamine receptor, which was reported to be reduced in RLS, decreased with the increase of age [40, 41]. Late-onset RLS patients showed stronger association with serum iron status and less decrease in brain iron compared to early-onset RLS patients [42, 43]. Complex action of pathogenic mechanisms according to age might influence on the association between 2 disorders in elderly.

Second, RLS is positively associated with many medical conditions such as neuropathy, radiculopathy, renal failure, iron-deficiency anaemia, cardiac diseases, genitourinary diseases, gastrointestinal diseases, and pulmonary diseases [44]. If associated with 1 of these known contributors, RLS is considered to be secondary to the condition. Considering that the prevalence of diseases that are related to RLS could be elevated in the elderly population, it may be the case that the secondary RLS prevalence is also increased. A general increase in secondary RLS and decrease in the migraine prevalence within the elderly population could induce the observed lack of an association between migraine and RLS. We did not attempt to evaluate other medical conditions which were related to secondary RLS in the present study. As such, the results of this study have a limitation regarding secondary RLS.

Third, some medications such as neuroleptic agents, antidepressants, opioid antagonists, and antiemetic agents can cause or exacerbate RLS and cease or relieve migraine [45, 46]. Elderly populations are more likely to take these medications and that could result in a higher RLS prevalence within the elderly group without altering the migraine prevalence. This medication-induced increase in the RLS prevalence and decrease in the migraine prevalence in the elderly group may also result in the observed lack of an association between migraine and RLS in the elderly group.

Previous studies regarding the association between non-migraine headaches and RLS has been reported in clinic-based setting [23, 47–51]. Some studies reported positive results regarding the association between non-migraine headaches and RLS but other studies reported negative results. Our study firstly investigated the association between non-migraine headaches and RLS in a population-based setting and found a positive result. A recent population-based study reported a positive association between RLS and multi-site body pain [52]. Further population-based studies regarding the association between non-migraine headaches and RLS would be needed to verify the association between non-migraine headaches and RLS.

Prior to the current work, RLS prevalence data among migraineurs according to migraine/headache status were not available in a population-based study. Our study showed that RLS was more prevalent among migraineurs with 1–10 attacks per month than among migraineurs with <1 attack per month (Table 3). The HIT-6 score of migraineurs with RLS was greater than that of migraineurs without RLS (59.1 ± 9.4 vs. 53.7 ± 9.2, p = 0.047). Considering headache frequency and HIT-6 score are important markers for migraine severity, these findings may corroborate the association between migraine and RLS.

Our study has several limitations. First, we diagnosed RLS based on participants’ reports according to IRLSSG criteria and some conditions similar to RLS may be included. It is known that these criteria can mimic other conditions including nocturnal leg cramps, anxiety disorders, akathisia, meralgia paresthetica, and peripheral neuropathies [44]. To minimise the accidental inclusion of these conditions, a new diagnostic questionnaire was recently proposed [53]. It showed a high specificity but has not yet been used in an epidemiological study. Second, we did not assess the severity of RLS due to limits on the questionnaire length [54]. An attempt to model a quantitative correlation between migraine and RLS may add better insights to the association between these 2 disorders. As such, further study including the RLS severity scale would be needed to better understanding this association. Third, although this is a population-based study with a low sampling error, its statistical power for examining subgroups was limited. Thus, some results might have not reached statistical significance merely because of the limited sample numbers, especially for small groups based upon the age-band. Fourth, we did not thoroughly investigate the secondary causes of headache because this is difficult to document with the questionnaire method used in this population study. Although most of recurrent headache sufferers in general population were considered to have primary headaches rather than secondary headaches, some secondary headaches sufferers might be classified as having primary headaches [55]. The 1-year prevalences of all headaches and migraine were similar to previous studies in Korean and other Asian countries [3, 6, 7, 34, 56].

Our study has several strengths. First, we used clustered random sampling proportional to the Korean population and the estimated sampling error was low. Second, we used the ICHD-2 and IRLSSG criteria for the diagnosis of migraine and RLS, respectively. Third, we analysed RLS prevalence according to headache frequency among migraineurs. Fourth, we assessed the association between non-migraine headaches and RLS in a population-based setting. Balancing the limitations and the strengths, we think that this study successfully assessed the association between migraine and RLS according to age.

## Conclusions

In summary, migraine and non-migraine headaches showed positive associations with RLS in adults aged 19–49 but failed to show any association in adults aged 50–69 in the present study. The RLS prevalence was higher among migraineurs with ≥1 headache attacks per month compared to migraineurs with <1 headache attacks per month. This information may provide a better understanding regarding the comorbidity and pathophysiology of migraine, headache, and RLS.

### Ethical standards

The study was approved by the institutional review board/ethics committee of Hallym University Sacred Heart Hospital and was performed in accordance with the ethical standards laid down in the 1964 Declaration of Helsinki and its later amendments. Written informed consent was obtained from all participants.

## Additional file

Additional file 1:
**Questionnaire in English.**

## Abbreviations

CI: Confidence interval
HIT-6: Headache Impact Test-6
ICHD-2: The second edition of International Classification of Headache Disorders
IRLSSG: International Restless Legs Syndrome Study Group
OR: Odds ratio
PSQI: Pittsburgh Sleep Quality Index
RLS: Restless legs syndrome
VAS: Visual analogue scale

## References

[1] Bigal ME, Lipton RB (2009). The epidemiology, burden, and comorbidities of migraine. Neurologic Clinics.

[2] Hu XH, Markson LE, Lipton RB, Stewart WF, Berger ML (1999). Burden of migraine in the United States: disability and economic costs. Archives of Internal Medicine.

[3] Kim B-K, Chu MK, Lee TG, Kim J-M, Chung C-S, Lee K-S (2012). Prevalence and impact of migraine and tension-type headache in Korea. Journal of Clinical Neurology.

[4] Leonardi M, Steiner TJ, Scher AT, Lipton RB (2005). The global burden of migraine: measuring disability in headache disorders with WHO's Classification of Functioning, Disability and Health (ICF). The Journal of Headache and Pain.

[5] Lipton RB, Stewart WF, Diamond S, Diamond ML, Reed M (2001). Prevalence and burden of migraine in the United States: data from the American Migraine Study II. Headache.

[6] Sakai F, Igarashi H (1997). Prevalence of migraine in Japan: a nationwide survey. Cephalalgia.

[7] Wang SJ, Fuh JL, Young YH, Lu SR, Shia BC (2000). Prevalence of migraine in Taipei, Taiwan: a population‐based survey. Cephalalgia.

[8] Steiner TJ, Stovner LJ, Birbeck GL (2013). Migraine: the seventh disabler. Cephalalgia.

[9] Breslau N, Lipton R, Stewart W, Schultz L, Welch K (2003). Comorbidity of migraine and depression investigating potential etiology and prognosis. Neurology.

[10] Scher AI, Bigal ME, Lipton RB (2005). Comorbidity of migraine. Current Opinion in Neurology.

[11] Buse DC, Rupnow MF, Lipton RB (2009). Assessing and managing all aspects of migraine: migraine attacks, migraine-related functional impairment, common comorbidities, and quality of life. Mayo Clinic Proceedings.

[12] Lipton RB, Silberstein SD (1994) Why study the comorbidity of migraine? Neurology 44(10 Suppl 7):S4–S5 7969946

[13] Diamond ML (2002). The role of concomitant headache types and non-headache co-morbidities in the underdiagnosis of migraine. Neurology.

[14] Allen RP, Picchietti D, Hening WA, Trenkwalder C, Walters AS, Montplaisi J (2003). Restless legs syndrome: diagnostic criteria, special considerations, and epidemiology: a report from the restless legs syndrome diagnosis and epidemiology workshop at the National Institutes of Health. Sleep Medicine.

[15] Ohayon MM, O’Hara R, Vitiello MV (2012). Epidemiology of restless legs syndrome: a synthesis of the literature. Sleep Medicine Reviews.

[16] Cho S-J, Hong JP, Hahm B-J, Jeon HJ, Chang SM, Cho MJ, Lee HB (2009). Restless legs syndrome in a community sample of Korean adults: prevalence, impact on quality of life, and association with DSM-IV psychiatric disorders. Sleep.

[17] Cho YW, Shin WC, Yun CH, Hong SB, Kim JH, Allen RP, Earley CJ (2008). Epidemiology of restless legs syndrome in Korean adults. Sleep.

[18] Kim J, Choi C, Shin K, Yi H, Park M, Cho N, Kimm K, Shin C (2005). Prevalence of restless legs syndrome and associated factors in the Korean adult population: the Korean Health and Genome Study. Psychiatry and Clinical Neurosciences.

[19] Kim WH, Kim BS, Kim SK, Chang SM, Lee DW, Cho MJ, Bae JN (2012). Restless legs syndrome in older people: a community‐based study on its prevalence and association with major depressive disorder in older Korean adults. International Journal of Geriatric Psychiatry.

[20] Lee M-J, Kwon OD, Kim J-E (2012). Epidemiologic Study of Restless Leg Syndrome in Rural Elderly; The Interview Research on Unsu-Myun. J Korean Sleep Res Soc.

[21] Ma J-F, Xin X-Y, Liang L, Liu L-H, Fang R, Zhang Y-J, Wang D-Y, Fahn S, Tang H-D, Chen S-D (2012). Restless legs syndrome in Chinese elderly people of an urban suburb in Shanghai: a community-based survey. Parkinsonism & Related Disorders.

[22] Nomura T, Inoue Y, Kusumi M, Uemura Y, Nakashima K (2008). Prevalence of restless legs syndrome in a rural community in Japan. Movement Disorders.

[23] Chen P-K, Fuh J-L, Chen S-P, Wang S-J (2010). Association between restless legs syndrome and migraine. Journal of Neurology, Neurosurgery & Psychiatry.

[24] Schürks M, Winter A, Berger K, Kurth T (2014) Migraine and restless legs syndrome: A systematic review. Cephalalgia:0333102414537725 10.1177/0333102414537725 25142142

[25] Schürks M, Winter AC, Berger K, Buring JE, Kurth T (2012). Migraine and restless legs syndrome in women. Cephalalgia.

[26] Suzuki S, Suzuki K, Miyamoto M, Miyamoto T, Watanabe Y, Takashima R, Hirata K (2011). Evaluation of contributing factors to restless legs syndrome in migraine patients. Journal of Neurology.

[27] Winter AC, Schürks M, Berger K, Buring JE, Gaziano JM, Kurth T (2012) Migraine and restless legs syndrome in men. Cephalalgia:0333102412466965 10.1177/0333102412466965 PMC3528814 23155191

[28] Zanigni S, Giannini G, Melotti R, Pattaro C, Provini F, Cevoli S, Facheris MF, Cortelli P, Pramstaller PP (2014). Association between restless legs syndrome and migraine: a population‐based study. European Journal of Neurology.

[29] Park JW, Moon HS, Kim JM, Lee KS, Chu MK (2014). Chronic daily headache in Korea: prevalence, clinical characteristics, medical consultation and management. J Clin Neurol.

[30] Oh K, Cho S-J, Chung YK, Kim J-M, Chu MK (2014). Combination of anxiety and depression is associated with an increased headache frequency in migraineurs: a population-based study. BMC Neurology.

[31] Headache Classification Subcommittee of the International Headache Society (2004). The International Classification of Headache Disorders: 2nd edition. Cephalalgia.

[32] Sohn SI, Kim DH, Lee MY, Cho YW (2012). The reliability and validity of the Korean version of the Pittsburgh Sleep Quality Index. Sleep and Breathing.

[33] Rubin D, Little R (2002). Statistical analysis with missing data.

[34] Stovner L, Hagen K, Jensen R, Katsarava Z, Lipton R, Scher A, Steiner T, Zwart JA (2007). The global burden of headache: a documentation of headache prevalence and disability worldwide. Cephalalgia.

[35] Peng KP, Wang SJ (2014). Epidemiology of Headache Disorders in the Asia‐Pacific Region. Headache.

[36] MacGregor EA, Rosenberg JD, Kurth T (2011). Sex‐Related Differences in Epidemiological and Clinic‐Based Headache Studies. Headache.

[37] Allen R (2004). Dopamine and iron in the pathophysiology of restless legs syndrome (RLS). Sleep Medicine.

[38] Charbit AR, Akerman S, Goadsby PJ (2010). Dopamine: what's new in migraine?. Current Opinion in Neurology.

[39] Kruit MC, Launer LJ, Overbosch J, Van Buchem MA, Ferrari MD (2009). Iron accumulation in deep brain nuclei in migraine: a population‐based magnetic resonance imaging study. Cephalalgia.

[40] Antonini A, Leenders KL, Reist H, Thomann R, Beer H-F, Locher J (1993). Effect of age on D2 dopamine receptors in normal human brain measured by positron emission tomography and 11C-raclopride. Archives of Neurology.

[41] Connor JR, Wang X-S, Allen RP, Beard JL, Wiesinger JA, Felt BT, Earley CJ (2009). Altered dopaminergic profile in the putamen and substantia nigra in restless leg syndrome. Brain.

[42] Allen RP, Earley CJ (2000). Defining the phenotype of the restless legs syndrome (RLS) using age-of-symptom-onset. Sleep Medicine.

[43] Earley CJ, Barker PB, Horská A, Allen RP (2006). MRI-determined regional brain iron concentrations in early-and late-onset restless legs syndrome. Sleep Medicine.

[44] Benes H, Walters AS, Allen RP, Hening WA, Kohnen R (2007). Definition of restless legs syndrome, how to diagnose it, and how to differentiate it from RLS mimics. Movement Disorders.

[45] Hoque R, Chesson AL (2010). Pharmacologically induced/exacerbated restless legs syndrome, periodic limb movements of sleep, and REM behavior disorder/REM sleep without atonia: literature review, qualitative scoring, and comparative analysis. Journal of Clinical Sleep Medicine.

[46] Loder E, Burch R, Rizzoli P (2012). The 2012 AHS/AAN guidelines for prevention of episodic migraine: a summary and comparison with other recent clinical practice guidelines. Headache.

[47] Cetinkaya Y, Yilmaz NC, Turkoglu R, Gencer M, Tireli H (2009). The relationship between tension-type headache patients with anemia and restless-leg syndrome. Journal of Neurological Sciences (Turkish).

[48] d’Onofrio F, Bussone G, Cologno D, Petretta V, Buzzi MG, Tedeschi G, Bonavita V, Cicarelli G (2008). Restless legs syndrome and primary headaches: a clinical study. Neurological Sciences.

[49] Daniela C, Giulio C, Vittorio P, Gabriella M, Vincenzo T, Eliana M, Gerardo C, Gennaro B (2011). Cluster headache patients are not affected by restless legs syndrome: an observational study. Clinical Neurology and Neurosurgery.

[50] Rozen TD, Fishman RS (2012). Cluster Headache in the United States of America: Demographics, Clinical Characteristics, Triggers, Suicidality, and Personal Burden. Headache.

[51] Young WB, Piovesan EJ, Biglan KM (2003). Restless legs syndrome and drug-induced akathisia in headache patients. CNS Spectrums.

[52] Stehlik R, Ulfberg J, Hedner J, Grote L (2014). High prevalence of restless legs syndrome among women with multi‐site pain: A population‐based study in Dalarna, Sweden. European Journal of Pain.

[53] Allen RP, Burchell BJ, MacDonald B, Hening WA, Earley CJ (2009). Validation of the self-completed Cambridge-Hopkins questionnaire (CH-RLSq) for ascertainment of restless legs syndrome (RLS) in a population survey. Sleep Medicine.

[54] Restless Legs Syndrome Study Group (2003). Validation of the International Restless Legs Syndrome Study Group rating scale for restless legs syndrome. Sleep Medicine.

[55] Rasmussen BK, Jensen R, Schroll M, Olesen J (1991). Epidemiology of headache in a general population—a prevalence study. Journal of Clinical Epidemiology.

[56] Wang S-J (2003). Epidemiology of migraine and other types of headache in Asia. Current Neurology and Neuroscience Reports.

