# Supplementary material for: Migraine and restless legs syndrome are associated in adults under age fifty but not in adults over fifty: a population-based study
Source: J Headache Pain. 2015 Aug 13;16:75. doi: 10.1186/s10194-015-0554-0 (PMC4536239; doi:10.1186/s10194-015-0554-0)
Supplement: Additional file 1: — Questionnaire in English. [file 10194_2015_554_MOESM1_ESM.docx]

Basic personal information

Gender: (men/ women)

Residential area: (si)/(gun)

Size of residential area

(1) Large cities (2) Small or middle cities (3) Countryside

1. How old are you? years old
2. When were you born in the year of 19
3. What is your age group?

(1) 19-29 years old

(2) 30-39 years old

(3) 40-49 years old

(4) 50-59 years old

(5) 60 years or more

4. In the past year, have you had at least one headache lasting more than 1 minute?

(Yes)/ (No)

These questions about your “most bothersome headache” during the previous 1 year (No. 5-25)

1. On average, how long did these headaches last?

( ) second(s), ( ) minute(s), ( ) hour(s), ( ) day(s),

6. How often did you experience such headaches during the last 1 year?

Daily ( times), weekly ( time), monthly ( times), yearly ( times),

7. How bad was your headache?

(1) Headache did not disturb usual daily activities (mild).

(2) Headache often disturbed usual daily activities, but I could perform more than half of my daily activities (moderate).

(3) I can’t perform my usual daily activities when I suffer these headaches (severe)

8. How severe was your headache? Please indicate a mark on the line which displays most properly about intensity of your headache. (O is no pain state and 10 is worst possible pain state)

| **통증이**  **없음** | |  | | | | | |  | | | | | |  | | | | | | **가장**  **심한 통증** | |
| --- | --- | --- | --- | --- | --- | --- | --- | --- | --- | --- | --- | --- | --- | --- | --- | --- | --- | --- | --- | --- | --- |
|  |  |  | | | | | |  | | | | | |  | | | | | |  |  |
|  |  |  |  |  |  |  |  |  |  |  |  |  |  |  |  |  |  |  |  |  |  |
|  |  |  |  |  |  |  |  |  |  |  |  |  |  |  |  |  |  |  |  |  |  |
| 0 | | 1 | | 2 | | 3 | | 4 | | 5 | | 6 | | 7 | | 8 | | 9 | | 10 | |

9. What was the location of the headache?

(1) Right side (2) Left side (3) Bilateral side (4) whole head

(5) Unilateral either way (6) Here and there (migrating)

10. What was the headache like? Please describe your headache properly.

11. What was the headache like? Please statement all describes your headache during the previous year.

(1) Pulsating and throbbing

(2) Heavy and stiff

(3) Tightening feeling like tying a band around your head

(4) Sharp like pinpricking

(5) Sudden and severe like hitting your head with a hammer

(6) Other: describe

These are questions asking about your headaches. (No. 12-24)

| Question No. |  | Yes | No |
| --- | --- | --- | --- |
| 12 | Do you feel sick to your stomach during your headaches? |  |  |
| 13 | Do you feel nauseated during your headaches? |  |  |
| 14 | Do you vomit during your headaches? |  |  |
| 15 | Do light bother you a lot more than when you don’t have headaches? |  |  |
| 16 | The headache worsened by activities such as walking or climbing stairs? |  |  |
| 17 | Is your headache more painful when you are in noisy surroundings? |  |  |
| 18 | Do you feel differently or uncomfortable smell sense than you don’t have headaches? |  |  |
| 19 | Do you see scintillating light, glittering stars or experience blurring of vision before or during your headaches? |  |  |
| 20 | Did you feel dizzy sense before or during your headaches? |  |  |
| 21 | Did you experience a sudden severe headache? |  |  |
| 22 | Did you experience unilateral headaches, presenting less than 4 h in a day, for more than 7 days? |  |  |
| 23 | Did you miss activities in work, school or house shores by headache during the previous 3 months? |  |  |
| 24 | Did you experience decreased activities in work, school or house shores by headache during the previous 3 months? |  |  |

25. If you experienced decreases activity or missed activity in work, school or house shores, How many days did you experience decreased activity or missed activity days in work, school or house shores during the previous 3 months

1. Missed activity days days

2. Decreased activity days days

(Do not include days you counted in question 1 where you missed activity in work, school or house shore)

26. Have you ever visit medical doctor(s) for your headache? (Yes) / (No)

27. If you have visited medical doctor(s) for headache, what type(s) of doctors have you visited? Please check all you have visited

(1) Neurologist

(2) Internal medicine doctor

(3) Neurosurgeon

(4) Family doctor

(5) Dentist

(6) Other medical doctor

(7) I did not know the type of the doctor

28-1. If you have visited doctor(s) for your headache, Have you ever heard of the diagnosis of your headache from doctor(s)? (Yes) / (No)

28-2. What was your diagnosis of your headache?

29. How do you treat your headache? Please check all treatment which you had received during the previous 1 year.

(1) Not treated

(2) Acupuncture

(3) Herbal medicine at oriental medical clinic

(4) Over the counter medications

(5) Prescription drugs

(6) Others

30-1. If you take medication(s) for headaches, how often did you take medication for your headache during the previous year?

(1) ( ) times in a year

(2) ( ) times in a month

(3) ( ) times in a week

(4) ( ) times in a day

(5) ( ) times in an hour

30-2. What is your most commonly administering drug for your headache? Please describe .

31 . If you take prescripton drugs for your headache, how did you take it?

(1) I visit doctor for headache and take medication when I had a severe headache

(2) I visit doctors regularly and take prescription drugs only when I have a headache

(3) I visit doctors regularly and take drugs to prevent headache

These questions are about your headache. Please chose the way you feel and what you can not do because of your headaches. (No. 32-1~32-6)

| 32-1 | When you have headaches, how often is the pain severe? | | | | |
| --- | --- | --- | --- | --- | --- |
|  | Never | Rarely | Sometimes | Very Often | Always |
| 32-2 | How often do headaches limit your ability to do usual daily activities including household work, work, school, or social activities? | | | | |
|  | Never | Rarely | Sometimes | Very Often | Always |
| 32-3 | When you have a headache, how often do you wish you could lie down? | | | | |
|  | Never | Rarely | Sometimes | Very Often | Always |
| 32-4 | In the past 4 weeks, how often have you felt too tired to do work or daily activities because of your headaches? | | | | |
|  | Never | Rarely | Sometimes | Very Often | Always |
| 32-5 | In the past 4 weeks, how often have you felt fed up or irritated because of your headaches? | | | | |
|  | Never | Rarely | Sometimes | Very Often | Always |
| 32-6 | In the past 4 weeks, how often did headaches limit your ability to concentrate on work or daily activities? | | | | |
|  | Never | Rarely | Sometimes | Very Often | Always |

33. What is your profession?

1. Farmers, fishermen or other primary employment
2. Self-employment
3. Sales/ service
4. Laborer
5. Other blue colors
6. Office worker
7. Administrative workers
8. Expert or specialized job
9. House wife
10. Student
11. Unemployed
12. Other profession: describe

34. What is your family’s approximate monthly income ?

(1) Less than 490,000 KRW (Korean won) (2) 500,000-990,000 KRW

(3) 1,000,000-1,490,000 KRW (4) 1,500,000-1,990,000 KRW

(5) 2,000,000-2,490,000 KRW (6) 2,500,000-2,990,000 KRW

(7) 3,000,000-3,490,000 KRW (8) 3,500,000-3,990,000 KRW

(9) 4,000,000-4,490,000 KRW (10) 5,000,000-5,990,000 KRW

(11) 6,000,000-6,990,000 KRW (12) More than 7,000,000 KRW

35. How much schooling have you had?

(1) Elementary school graduated or less

(2) Middle school graduated

(3) High school graduated

(4) College graduated or college student

(5) Graduate school graduated or Graduate school student
